# Isolation of Novel *Mycobacterium* Species from Skin Infection in an Immunocompromised Person

**DOI:** 10.3201/eid2711.210426

**Published:** 2021-11

**Authors:** You-Ming Mei, Qian Zhang, Wen-Yue Zhang, Hai-Qin Jiang, Ying Shi, Jing-Shu Xiong, Le Wang, Yan-Qing Chen, Si-Yu Long, Chun Pan, Gai Ge, Zhen-Zhen Wang, Zi-Wei Wu, Yan Wang, Yi-Qun Jiang, Hong-Sheng Wang

**Affiliations:** Chinese Academy of Medical Sciences and Peking Union Medical College, Institute of Dermatology, Nanjing, China

**Keywords:** antimicrobial resistance, bacteria, China, Mycobacterium camsnse, Mycobacterium gordonae, nontuberculous mycobacteria infection, skin infections, tuberculosis and other mycobacteria, whole-genome sequencing

## Abstract

We investigated a case of cutaneous infection in an immunocompromised patient in China that was caused by a novel species within the *Mycobacterium gordonae* complex. Results of whole-genome sequencing indicated that some strains considered to be *M. gordonae* complex are actually polyphyletic and should be designated as closely related species.

*Mycobacterium gordonae* was first described 50 years ago as a slow-growing scotochromogenic nontuberculous mycobacterium. Previous research revealed vague molecular typing results for *M. gordonae*–like strains. For example, the RNA polymerase-β (*rpoB*) PCR restriction analysis discriminates *M. gordonae* into 4 clusters even though cluster D does not hybridize well with the type strain (*1*). Two novel species, *M. paragordonae* and *M. vicinigordonae*, share >99% 16S rRNA gene similarity with *M. gordonae*, which might also lead to confusion about their classification (*2*,*3*).

*M. gordonae* is frequently isolated from water systems and clinical samples as a potential opportunistic pathogen (*4*,*5*); clinical infections ranging from skin and lung infections to disseminated systemic infections have been reported, especially in immunosuppressed patients (*6*,*7*). Both the *M. paragordonae* and *M. vicinigordonae* strains were first isolated as nonpathogenic organisms from pneumonia patients (*2*,*3*). *M. paragordonae* is often isolated from hospital water systems and devices, but only 1 case of iatrogenic *M. paragordonae* infection has been reported (*8*,*9*). These reports reveal the dissimilar effects produced by different *M. gordonae*–like strains.

The advent of whole-genome sequencing has brought genomewide analyses into common use to delineate species (*10*–*12*). The widely accepted cutoffs adopted for the average nucleotide identity (ANI) and in silico DNA–DNA hybridization (isDDH), 95%–97% for ANI and 70% for isDDH, strongly correlate with traditional DDH division values, providing more robust resolution than phenotyping or mycolic acid analysis for determining mycobacterial taxonomy (*11*,*12*). We report a case of cutaneous infection in Jiangsu Province, China, caused by a previously undescribed novel species belonging to the *M. gordonae* group.

## The Study

**Figure 1:**
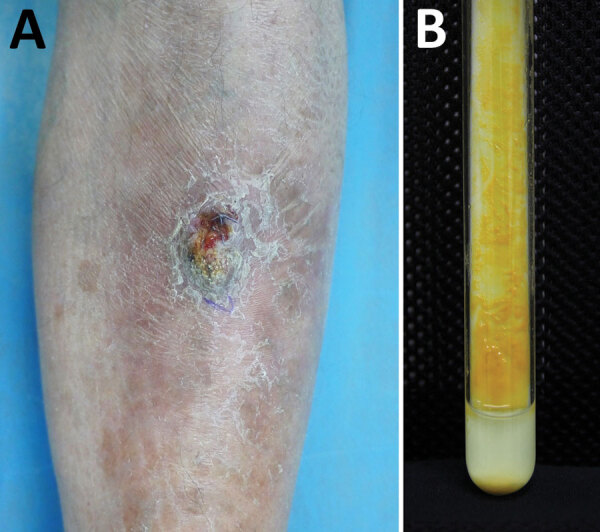
Novel *Mycobacterium gordonae*–like infection in a 63-year-old man in China. A) Verrucous dull nodule on the left shin of the patient. B) *Mycobacterium* colonies grown on Löwenstein–Jensen medium.

A 63-year-old man was admitted to the hospital for a 5-year history of a nodule on his left shin. The asymptomatic lesion initially appeared as a papule and gradually developed into a dull red verrucous nodule with scales (Figure 1, panel A). No trauma history before the onset was reported. The patient had received a diagnosis of lupus erythematosus 30 years earlier and had taken oral prednisone (20 mg/d) over the previous year. Laboratory test results indicated no remarkable findings. Histologic examination of a skin sample showed irregular epithelial hyperplasia and granulomatous infiltrations of a large number of epithelioid histocytes, neutrophil cells, plasma cells, and lymphocytes in the dermal layer. After 19 days of culture, orange colonies were observed on modified Löwenstein–Jensen slants at 32°C (Figure 1, panel B). The organism was scotochromogenic with a smooth appearance and grew well at 32°C and 37°C on both Löwenstein–Jensen slants and Middlebrook 7H9 with oleic acid dextrose citrate. The colonies were confirmed to be rod-shaped, acid-fast bacterium.

We extracted DNA from the colonies for PCR analysis and compared the sequences using BLAST (https://blast.ncbi.nlm.nih.gov/Blast.cgi). The 16S rRNA (1452 bp) gene shared greatest similarity (99.51%) with *M. gordonae* *ASCr-1.2*; gene sequencing showed *rpoB* (365 bp) shared 97.53% and *hsp65* (765 bp) 95.53% similarity with *M. paragordonae* 49061. On the basis of these results, we diagnosed infection with a member of the *M. gordonae* complex. Drug sensitivity analysis revealed the bacterium to be sensitive to moxifloxacin, levofloxacin, ethambutol, and amikacin and resistant to clarithromycin, isoniazid, and rifampicin; we therefore prescribed a moxifloxacin regimen for the patient. Because the lesion had not healed over several years, we surgically resected it and applied photodynamic therapy after 2 months of antimicrobial drug treatment. One month later, the patient reported that the lesion had recovered well and refused further oral antimicrobial drugs. No recurrence was observed in the following year.

To accurately identify the pathogen to the species level, we performed whole-genome sequencing (8,509,558 reads, 110×) of the isolate X7091 using the Illumina Hiseq 4000 (https://www.illumina.com) and PacBio RS II (https://www.pacb.com) platforms at the Beijing Genomics Institute. Sequence data indicated a 7.1-Mb genome (7,319,570 bp) including a plasmid (216,348 bp) with a guanine-cytosine content of 64.6% (Genbank accession no. GCA_017086405.1). The complete genome had a guanine-cytosine content of 66.7%, similar to *M. gordonae* (66.8%) and *M. paragordonae* (67.0%). Functional annotation obtained through multiple databases revealed 6,704 coding sequences, 48 tRNA, 3 rRNA, and 35 small RNA genes.

We compared this isolate with all available genomes of the *M. gordonae* group using whole-genome-based computational strategies. ANI calculated by FastANI (https://github.com/ParBLiSS/FastANI) revealed that the closest matches, with *M. gordonae* HMC_M15 (87.80%) and *M. gordonae* DSM 44160 (87.79%), were well below the threshold for species delineation (Appendix Table) (*11*). Evaluating isDDH using the Type Strain Genome Server (https://tygs.dsmz.de) showed weak relations with *M. gordonae* DSM 44160 (34.5%) and *M. paragordonae* 49061 (31.3%) (*13*) (Appendix Table); we found no closely related genome in the database. For *M. paragordonae* strains, ANI was 97.8%–98.6% and isDDH 80.4%–99.9%; for *M. gordonae* strains, ANI was 99.1%–99.9% and isDDH 93.0%–99.3%. 

**Figure 2:**
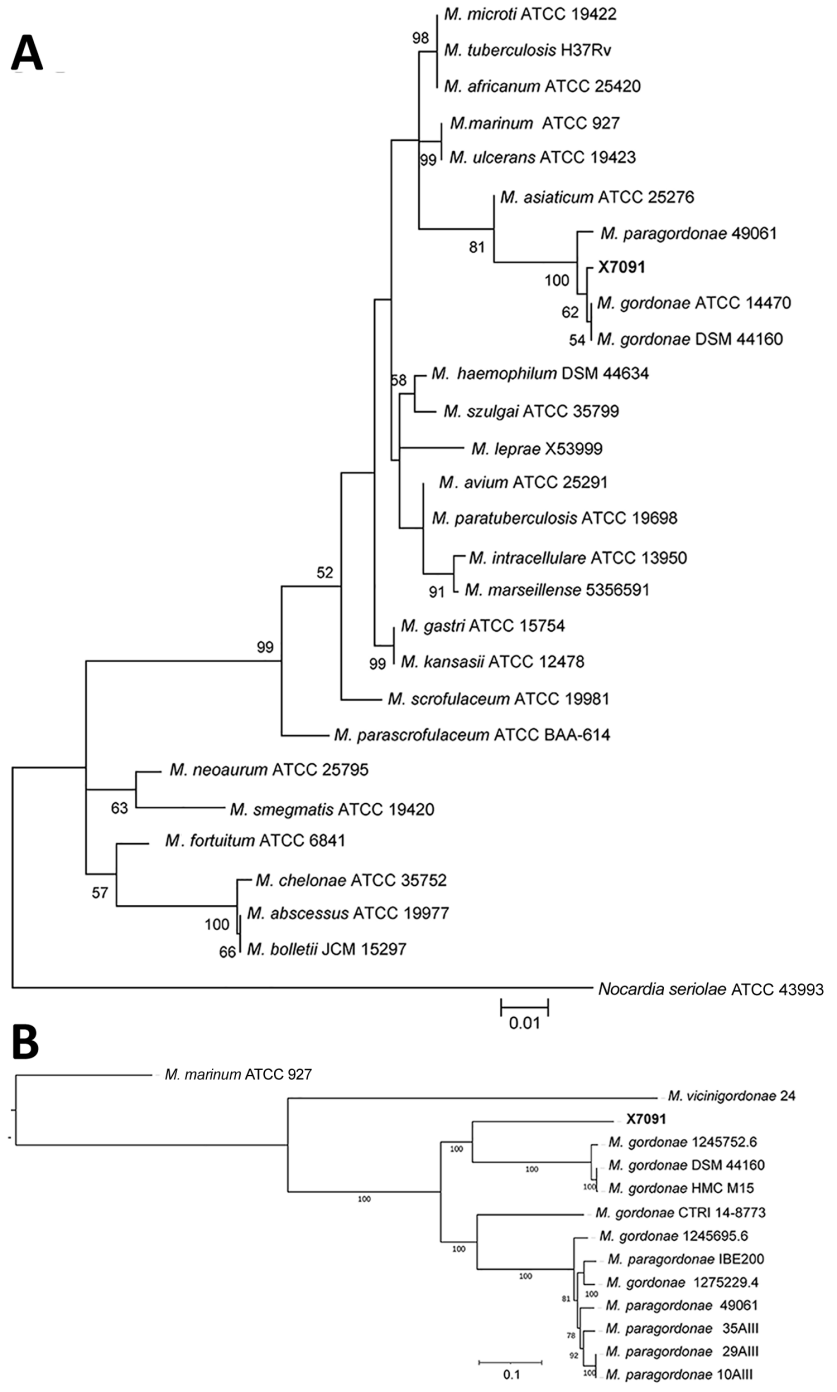
Phylogenetic trees of isolate from novel *Mycobacterium gordonae*–like infection in a 63-year-old man in China (X7091) and reference isolates. A) Evolutionary tree involving *16S rRNA* gene (1,067 positions) of isolate X7091 and 26 *Mycobacterium* strains. Tree constructed using the maximum-likelihood method and Tamura-Nei model with 500 bootstrap replications in MEGA X (https://www.megasoftware.net). We selected *Norcadia seriolae* ATCC 43993 as the outgroup. B) Core genome–based maximum-likelihood phylogeny of isolate X7091 and other *M. gordonae*–like strains analyzed by Roary (https://sanger-pathogens.github.io/Roary) and constructed with a general time-reversible plus gamma maximum model (500 bootstrap replications) using the RaxML tool (*14*). We selected *Mycobacterium marinum* ATCC 927 as the outgroup. Scale bars indicate the number of nucleotide substitutions per site.

The core-genome phylogeny of the *M. gordonae* complex, constructed using a previously described method, suggested that the isolated strain is a branch within the cluster but distant from other *M. gordonae*-like strains (*14*,*15*) (Figure 2, panel B). Integration of these highly concordant results strongly suggested that the isolate is distinct from present *M. gordonae*–like strains and represents a novel species within the *M. gordonae* complex. We proposed *Mycobacterium camsnse* sp. nov*.* as the name for this strain.

When comparing the similarity index of all available genomes of the *M. gordonae* group, we found clear demarcations among the *M. camsnse* X7091*, M. gordonae* CTRI 14-8773, *M. vicinigordonae* 24, 7 *M. paragordonae*, and 3 *M. gordonae* strains including the type strain DSM 44160 (Figure 2). Two strains recorded as *M. gordonae* ssp. in the cluster of *M. paragordonae* may have previously been misclassified. *M. gordonae* CTRI 14-8773, isolated in Russia, also represents a novel species of the *M. gordonae* group. These results confirmed the genomic diversity of *M. gordonae*–like strains, corroborating that the *M. gordonae* group is polyphyletic and should be divided into >5 closely related species.

## Conclusions

*M. gordonae* is generally considered a minimally pathogenic mycobacteria. Nonetheless, clinical infections have been reported, even in immunocompetent individuals (*6*–*7*). We isolated a distinct strain within the *M. gordonae* group from a skin infection using whole genome–level approaches based on ANI, isDDH, and core gene phylogeny. We proposed the name *Mycobacterium camsnse* sp. nov. for this strain. We also provided genomic insights into the heterogeneity of the *M. gordonae–*like strains, including finding 2 strains potentially misclassified as *M. gordonae* ssp., and demonstrated that the present *M. gordonae* group should be designated as 5 closely related species.

Conventional routines for describing species, such as wet-lab DDH and phenotypic tests, which are tedious and restricted by laboratory capacity, often do not delineate closely related species. Genome-based analysis affords a more accurate alternative for delineating species. Although digital gene-expression analyses might not have provided enough conclusive authentication data, the low genomewide similarity between the strain X7091 and *M. gordonae* group strongly support it as a novel species. Only a few *M. gordonae* assembly models are available, and large-scale investigations are needed to better understand species diversity, geographic distribution, and clinical significance of *M. camsnse* sp. nov. infections.

The persistent but limited nonpurulent lesion of the immunosuppressed patient in our study reflects the attenuated nature of the pathogen. The various drug resistance properties of X7091 and other *M. gordonae*–like strains indicate the need for drug sensitivity testing before initiating drug treatment for *M. gordonae–*like strain infections (*6*,*7*). Our patient responded well, indicating that operation combined with antimicrobial therapy could be a good option for treating environmental *Mycobacterium*-induced skin infections.

This study was supported by grants from the Chinese Academy of Medical Sciences Innovation Fund for Medical Sciences (2016-I2M-1-005, 2017-I2M-B&R-14) and the National Natural Science Foundation of China (81972950).

Appendix Additional information about infection from a novel species of *Mycobacteria gordonae* complex.
